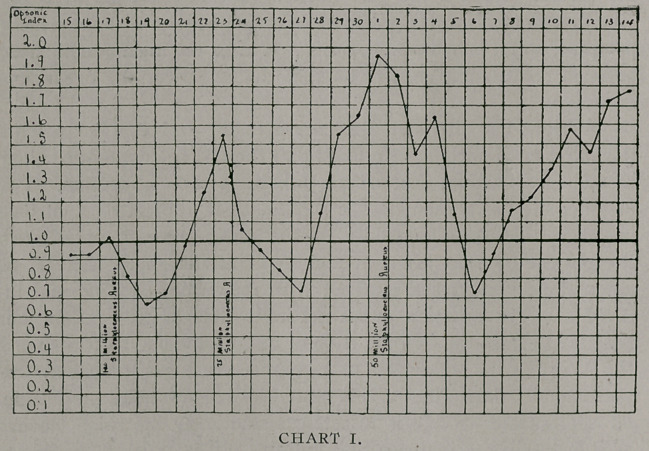# The Results of Vaccine Therapy in Acute and Chronic Infections

**Published:** 1909-01

**Authors:** J. Edgar Paullin

**Affiliations:** Atlanta, Ga.


### THE RESULTS OF VACCINE THERAPY IN ACUTE AND CHRONIC INFECTIONS.*

BY J. EDGAR PAULLIN, B. A., M. D., ATLANTA, GA.

Of the many infections to which the human body is ever exposed, the organisms most frequently concerned are the streptoccoccus, staphylococcus, gonococcus, pneumococcus, meningococcus, bacillus diphtheriae, bacillus typhosus, bacillus tuberculosis etc. As a result of the invasion by these organisms we have established in the body a process which may be either acute, chronic, localized or general. In the acute infections the clinical picture varies accordingly as the number and virulence of the infecting organism, being in many cases mild and without systematic disturbances, while in other cases there are the usual symptoms observed in a severe toxaemia. Acute infections run a rapid course and end in either recovery or death. Chronic infections, on the other hand, are marked by an insiduous onset without, as a rule, violent symptoms, and with a tendency toward very slow or practically no improvement.

Most infections are, however, localized, meaning by this that the invading organism is confined to some one particular area; in fact, it might he said that almost all infections are, in the beginning, localized and only become general on account of an inability of the body to successfully withstand the invasion of the bacteria so that they enter the blood stream, there multiply and are everywhere distributed throughout the body; producing the conditions commonly called septicaemia.

The portal of entry of these organisms is at a place far removed from the action of the blood fluids, or in that portion of the body which requires for its maintainence a comparatively small amount of blood or lymph. In other words, the organisms enter and multiply in an area of lowered bacteriotropic pressure.

In combatting the invading organisms, the body makes use of the protective substances normally present in the blood fluids—agglutinins, precipitins, lysins, opsonins, etc. Time does not permit a detailed statement concerning the action of each of these substances in protecting the body, yet it is generally believed that

*Read before Southern Medical Association, Atlanta, Ga.

a most important role is played in the protection of the body by the opsonin, and the amount of resistance offered to the invading organisms is proportional to the amount of opsonin present in the blood.

The work of Denys, Leclef and Mennes demonstrated that there is a substance in the blood which influences phagoctosis. Leishmann in 1902 published a simple method of estimating the amount of phagocytosis against the staphylococcus aureus in a patient the subject of this infection. It remained, however, for Wright to give experimental proof of the existence of a substance in the blood fluids which so act on bacteria as to cause them to be ingested by the phagocytes, and to show that the leukocytes when freed from blood serum possessed little or no phagocytic power. He showed that this substance, which so acts on bacteria as to render them capable of being ingested by the leukocytes, is present in the blood of normal individuals, and to it he gave the name of opsonin. About this time Neufeld and Rimpau made, independently, similar observations in working with anti-streptococcus serum. Later Opie and Barker have brought forward the fact that the leukocytes possess certain digestive ferments, leucoprotease, and it is by means of this that after the ingestion of these bacteria the enzyme so acts on them as to render the organisms harmless.

Wright has also called attention to the fact that in patients the subjects of chronic infections that the opsonic content of their blood is lower than that of an individual who is normal. For instance, he has shown that in a patient the subject of a chronic infection that the phagocytes of this patient when mixed with the patient's serum are not able to ingest as many bacteria as when the phagocytes are mixed with the serum of a normal individual. The question arises naturally then as to whether it is posisble to raise the opsonic content of the blood so as to increase the power of the leukocytes to ingest bacteria; and it has been experimentally shown that this is quite possible and takes place when an appropriate vaccine is injected; much after the same manner as it is possible to increase the agglutins. It is entirely possible to increase the opsonic power of the blood so that it will influence many times more bacteria than the blood of a normal individual.

A word might be said concerning vaccines; a vaccine is a sterilized and standardized culture of an organism. The method generally employed in making a vaccine is as follows: cultures are taken from the infected area and the nature of the infecting organism determined. Sub-cultures are then made and a twenty-four hour growth on agar is suspended in an 0.85 per cent. salt solution. The number of organisms in this solution are next counted by employing the procedure devised by Wright in which the suspension is compared to the blood of an individual containing a known number of red blood cells; equal parts of the suspension and blood are mixed, smears are made and the number of bacteria and red blood cells in several fields are counted. Knowing the number of red cells to the cm., it is easy to calculate the number of bacteria appearing in an equal amount of the bacterial suspension. Having determined the number of bacteria in the suspension, the vaccine is next sterilized by heating to 60 degrees Centrigrade for one hour. Sufficient carbolic acid is added to make it up to 0.1 per cent. Cultures are then made from this vaccine to be sure that it is sterile. In case it remains sterile, the vaccine is ready for use.

CHART I.

Following upon the injection of a vaccine, the condition of the blood is very instructive; shortly after the injection of the vaccine there is an appreciable decrease in the opsonins as evidenced by the fall in the curve—this is termed by Wright the "negative phase," and there is with this a decrease in the antibactericidal power of blood. In this chart the negative phase is quite marked on account of the fact that the initial dose of vaccine was large. Following upon the negative phase there is an increase in the antibactericidal substances of the blood as evidenced by an increase in the opsonic index; this has likewise been called by Wright the "positive phase." This positive phase may be maintained indefinitely by the regular administration of a vaccine. After the subsequent injections one observes that there is a drop in the measured opsonic blood content, but since the dose was reduced this negative phase was not so marked, neither did it last so long, and it rose higher after the previous injection. It is very easy to produce a series of negative phases on negative phases and by this means markedly decrease the patient's resistance to the invading organism by lowering the content of opsonin rendering the patient more succeptible to the action of the organism as is evidenced clinically by an aggravation of the condition about the infected area and an increase in its extent. On the other hand, it is just as easy to keep the index above normal provided one is familiar with the first signs of the negative phase as manifested by the clinical condition. The better way to be sure about this, to one who is not familiar with the negative phase clinically, is to determine the opsonic index, yet, as I have shown in a previous cmmunication (Journal-Record of Medicine, April, 1908), there are many objections to this procedure.

A word might here be said concerning the site of injection of the vaccine. It has appeared to me that the best results are obtained when the vaccine is administered at a point near the area of infection and at a point in the path of blood and lymph passing to the infected area. As to whether this matters at all or not, I am not as yet prepared to definitely state, but it seems as though some of my recent cases have demonstrated this to be true. The vaccine should in all cases be administered subcutaneously.

In the following cases I have used altogether for purposes of vaccination personal vaccines—meaning by this vaccines made from the particular organisms which were the cause of the infection in each particular case with the exception of the cases of tuberculosis. With these I have used three different vaccines: (1) Koch's old tuberculin, made from the human bacillus. (2) Koch's old tuberculin, made from the bovine bacillus. (3) Koch's bacillen emulsion. The doses of the various vaccines from the staphylococci have averaged from 10 to 100 million, depending upon the amount of resistance offered by the patient to each dose. The first dose has always been a small one, and has never been over 10 million organisms. In the use of tuberculin I generally give 1-100 of a milligram of Koch's old tuberculin as the starting dose, and occasionally give as high as 1-10 of a milligram over a space of time not exceeding three months. Of the bacillen emulsion the initial dose is 1-4,000 of a millegram of the dried tubercle bacilli, subsequently increasing the dose to as much as 1-500 of a milligram if necessary. I might state that the bacillen emulsion is composed of live tubercle bacilli which have been ground up, and, in order to be sure that no living organisms are present in this suspension, I heat this to 60 degrees Centigrade for one hour to be sure that the emulsion is sterile. I am convinced that the use of the bacillen emulsion gives better results than the other tuberculins mentioned. So in my cases now I am using only this.

CHART, NO. II.

| DISEASE. | Number of Cases | Well | Improved | Unimproved |
|---|---|---|---|---|
| Tuberculosis of skin -- -- -- | 2 | 1 | 1 | 0 |
| Tuberculosis of lung -- -- -- | 3 | 1 | 2 | 9 |
| Tuberculosis of bone -- -- -- -- | 3 | 0 | 2 | 1 |
| Tuberculosis of peritonaeum -- | 3 | 2 | 1 | 0 |
| Tuberculosis of lymph glands-- | 24 | 8 | 10 | 6 |
| Gonorrhoeal arthritis -- -- -- | 3 | 2 | 1 | 0 |
| Gonorrhoeal prostatis -- -- -- | 2 | 1 | 1 | 0 |
| Chronic furunculosis -- -- -- | 6 | 6 | 0 | 0 |
| Carbuncle -- -- -- -- -- -- -- -- | 2 | 2 | 0 | 0 |
| Chronic pustular acne-- -- -- | 10 | 6 | 3 | 1 |
| Chronic infection of knee joint | 1 | 0 | 1 | 0 |
| Sinus following empyema -- -- | 1 | 1 | 0 | 0 |
| Sycosis-- -- -- -- -- -- -- -- | 2 | 2 | 0 | 0 |
| Sinus following drainage of ab- | | | | |
| Streptococcus septicaemia -- -- | 1 | 1 | 0 | 0 |

Chart No. 2 shows the character of the cases which have been vaccinated. Of course, this has not been the only method used in the handling of these cases. Fresh air; sunshine; exercise; diet, together with local applications in some cases, have all been used. It is true that the number of cases is rather small, and no very definite conclusions can be drawn from them, but the hope is that enough interest will be aroused in this subject to give the work of Wright a more thorough trial.

In this chart all of my cases, including those now under treatment, are figured. The first of the cases is one of tuberculosis of the skin which had been treated by various local and general measures for a period of three years. In order to be sure of the diagnosis in this case a small piece of the skin was excised and examined microscopically, with the result that numbers of tubercles were found in the tissue, as well as tubercule bacilli. I immediately began injections of the bacillen emulsion, commencing with 1-2,000 of a milligram and going as high as 1-500 of a milligram. Those injections extended over a period of four months, at the end of which time the patient was practically well of the disease. In the beginning of this treatment several pockets which had burrowed beneath the skin were opened up and curetted by Dr. McRae. Ten months since patient received last injection, result perfect.

Three cases of tuberculosis of the peritonaeum have come under my observation, a girl of 18 years, a man of 20, and a woman of 34. In these cases there was a moderate amount of fluid in the abdomen. They were given tuberculin with the result that they are, to all intents and purposes, practically well.

There are 24 cases of lymph gland tuberculosis. Of these cases, 8 are well and 10 are improved. Included in the 10, there are 7 cases which have been under observation less than two months, and one case under observation ten months. Of the 8 cases well, these were under treatment from 4 to 8 months. One of these cases was discharged over a year ago and has remained perfectly well since. Of the 6 cases charted as unimproved, one has been under observation three months; one, a negro girl, who depended absolutely upon weekly injections of tuberculin to cure her—utterly disregarding the other measures advised; four cases have received only three doses of tuberculin, consequently little improvement is to be expected in their condition. I would call particular attention to the fact that the improvement in some of the cases of tuberculosis of the lymph glands after two or three injections of vaccine is quite marked. In one case the patient complained of considerable pain in the neck, which disappeared almost completely after two injections of vaccine. It has been my observation that, particularly in cases of bone tuberculosis, there is almost always a cessation of the marked pain observed in these cases so soon as the vaccines are administered. As yet I have not had a sufficient number of patients with this particular disease to make a definite statement regarding this observation, but it has been a noticeable feature in those cases treated.

Of the three cases of gonorrheal arthritis, marked improvement was observed in two of the cases following the injection of the gonococcic vaccine., and in one case after the injection of 6 doses the patient refused to return for further treatment, giving as his reason that he was "perfectly well."

Another of these cases had a gonorrheal arthritis affecting the right knee. When seen, the condition had been present two months, in the meantime having been treated by various surgical procedures. When seen, he complained of great pain in the joint and there was a possible flexion of about 35 degrees. The tissue surrounding the joint was markedly infiltrated; quite hard and brawny to the touch. He was given 15 doses of the gonococcic vaccine. At the time when he passed from under observation he had a possible flexion of approximately 100 degrees and a complete cessation of pain. In this case, in conjunction with the vaccine, super-heated air and massage were employed.

There are six cases of furunculosis; two of these are physicians. In one there were 6 furuncles on the hand and 4 or 5 on the neck. This patient tells me that he observed marked improvement in his condition after the first injection of vaccine, in that the pain had diminished and within one week the boils had disappeared. This patient should have had another dose of vaccine, but he unfortunately had to return to his practice and could not wait the required time. As a result of this, one other furuncle returned after he reached home. The other cases recovered in three, four, five, ten and twelve weeks, respectively.

Ten cases of chronic pustular acne, of which 6 are perfectly well, 3 improved and 1 unimproved. Of the 2 improved, one of these cases has been under observation 4 months, the other 4 weeks. The unimproved case I am not able to satisfactorily explain, except for the fact that the young lady only had three small doses of vaccine. She decided that this aggravated the condition and refused to be further treated. Whether further injections would have benefitted her, I am unable to say. Of the ten cases, the staphylococcus aureus was isolated from 7; the staphylococcus citreus from 1, and the staphylococcus albus from 2. Those cases which are perfectly well were infected with the staphylococcus aureus in 5; staphylococcus citreus in 1, and staphylococcus albus in 1. These cases were under observation from three weeks to five months. Occasionally there is a slight return of the former condition in one of these patients, but this is immediately remedied by a small dose of the vaccine.

Of the two cases of sycosis here figured, one of them was of a duration of two years, the other seventeen months. In both cases the infecting organisms were the staphylococcus citreus. Vaccines were prepared, and within three months from the beginning of the treatment the patients were perfectly wll. In one, there was a slight return of the condition two months after being discharged, but a subsequent dose of the same vaccine immediately corrected this. These patients had been treated by local applications since the beginning of the disease.

An exceedingly interesting case was that of a sinus persisting from an old abscess in Pott's disease. In the same patient there was a sinus leading to the right ankle joint, the result of a tubercular infection. In this case cultures from these sinuses revealed the presence of the staphylococcus aureus, albus, and the colon bacillus. Vaccines were made from each of these organisms and injected into the patient. The sinus in the right ankle practically healed, but there was little improvement in the condition of the back further than the fact that the patient seemed to have less pain after the injections than previously, and there was a diminuation in the amount of the discharge.

One very interesting case of streptococcus septicaemia was observed in which the above organism was grown from the blood. The infection evidently occurred following an abortion. Within 48 hours after the injection of the first dose of vaccine, which was 5 million streptococci, the temperature began to fall and there was a general improvement in the patient's condition. In all, five doses of vaccine were administered, and the patient recovered satisfactorily. Whether this was one of thase curious cases that would have shown the same condition had the vaccine not been administered, I am not able to say, but I am inclined to believe that benefit was derived from this treatment.

### CONCLUSION.

The conclusions that seem warranted from the above related facts and the report of cases are:

(1) That by the injection of a vaccine it is possible to increase the opsonic content of the blood fluids, and by increasing the index increase the resistance of the patient to invading organisms.

(2) That a vaccine to have the most beneficial effect should be prepared from the organism causing the lesion.

(3) Care should be exercised in the injection of a vaccine in order to avoid the production of marked negative phases.

(4) That the site of injection should be in the path of the blood stream to the part affected.

(5) That the most marked benefit from the injection of vaccines seems to be in those cases suffering from chronic infections, and in those particular regions of the body where the lymph flow is diminished.

Before closing I wish to express my most hearty thanks to Drs. Floyd W. McRae, W. B. Armstrong, C. W. Strickler, Michael Hoke, C. R. Andrews, L. S. Hardin, W. A. Crowe, W. P. Nicholson, L. T. Pattillo, and J. H. Johns, for referring cases to me, and without whose help it would have been impossible for me to have reported the above series of cases.

RELEVANT LITERATURE.

1. Surle mecanisme de l'immunite chez le lapin vaccine' contre le streptococcus pyog. La Cellule, 1895, Vol. XI, p. 198.
2. Zeit. r. Hyg. 1897, Vol. XXV, p. 413.
3. Brit. Med. Jour. 1902, Vol. I, p. 73.
4. Proc. Royal Society, Vol. LXXII.
5. Proc. Royal Society, Vol. LXXIII.
6. Journal Exp. Medicine, Vol. VII, p. 316, Vol. IX, p. 207.
7. Jour. Inf. Diseases 1905, Vol. II, p. 128.
8. Proc. Royal Society, Vol. LXXIV.
9. Lancet 1905, Vol. II, p. 1603.
10. Deutsch. Med. Woch., Vol. XXX, p. 1458.
11. Zeit fur. Hyg. u Inf., Vol. LI, p. 283.
12. Archiv. de Medec. Exper. 1899, Vol. I.
13. Practitioner 1906, Vol. LXXVII, p. 64.
14. Jour. Inf. Diseases, 1906, Vol. III, p. 731.
15. Amer. Jour. Med. Sciences, Vol. CXXXII, p. 187, 175, 203.
16. Amer. Jour. Med. Sciences, Vol. CXXXIV, p. 175.
17. Jour. Amer. Med. Association, Vol. XLVI, p. 1407; Vol.

XLVII, p. 1721; Vol. XLVIII, p. 139, 571, 1255, 1570, 2181; Vol XLIX, p. 316, 479, 633, 1176, 1245, 1815.

18. Surgery, Gynaecology and Obstetrics, Vol. V, p. 373.

19. Johns Hopkins Hospital Bulletin, Vol. XVIII, p. 223, 232.

## Figures and Tables

**CHART I. f1:**